# Prevalence of *Cryptosporidium* Infections in Thailand and Its Association with HIV and Diarrhea: A Systematic Review and Meta-Analysis

**DOI:** 10.3390/medsci13030156

**Published:** 2025-08-26

**Authors:** Manas Kotepui, Aongart Mahittikorn, Jurairat Jongthawin, Chutima Rattanawan, Frederick Ramirez Masangkay

**Affiliations:** 1 Medical Technology Program, Faculty of Science, Nakhon Phanom University, Nakhon Phanom 48000, Thailand; 2 Department of Protozoology, Faculty of Tropical Medicine, Mahidol University, Bangkok 10400, Thailand; aongart.mah@mahidol.ac.th; 3 Faculty of Medicine, Mahasarakham University, Maha Sarakham 44000, Thailand; 4 Department of Medical Science, Amnatcharoen Campus, Mahidol University, Amnat Charoen 37000, Thailand; 5 Department of Medical Technology, Faculty of Pharmacy, University of Santo Tomas, Manila 1008, Philippines; 6 Research Center for the Natural and Applied Sciences, University of Santo Tomas, Manila 1008, Philippines

**Keywords:** *Cryptosporidium*, HIV, meta-analysis, protozoa, oocyst, Thailand

## Abstract

*Cryptosporidium* is a protozoan parasite and a major cause of diarrhea, especially in children and immunocompromised individuals. Despite evidence of its presence in Thailand, comprehensive national data remain limited. This systematic review and meta-analysis aimed to estimate the prevalence of *Cryptosporidium* infection in Thailand and assess its association with diarrhea, especially among vulnerable populations, to support targeted public health strategies. This systematic review followed the PRISMA guidelines and was registered with PROSPERO (CRD420251015538). A systematic search was conducted in PubMed, Embase, Scopus, Journals@Ovid, Nursing & Allied Health Premium, Google Scholar, and the Thai-Journal Citation Index (TCI) to identify studies on *Cryptosporidium* infections in humans in Thailand. Quality assessment was independently performed using Joanna Briggs Institute tools. Pooled prevalence and odds ratios (ORs) were estimated using random-effects meta-analyses. Heterogeneity was explored via meta-regression and subgroup analyses, and publication bias was assessed using funnel plots and Egger’s test. A total of 38 studies with 24,759 participants were included, mostly conducted in Central Thailand. The pooled prevalence of *Cryptosporidium* infections was 4.70% (95% CI: 2.68–8.13%), with the highest prevalence observed in Northern Thailand (11.14%) and among HIV-infected individuals (16.33%). Significant predictors of higher prevalence included region, age group, participant type, and diarrheal status. Infection was associated with a non-significant two-fold increased odds of diarrhea (*p*: 0.39; OR: 2.00; 95% CI: 0.67–5.99), but significantly higher odds of diarrhea among patients with *Cryptosporidium* infections were seen in the Central region (OR: 3.71; 95% CI: 1.17–11.8). HIV-seropositive individuals had a significantly higher risk of infection (*p*: 0.006; OR: 8.15; 95% CI: 1.82–36.50). This systematic review and meta-analysis estimated a pooled prevalence of *Cryptosporidium* infections in Thailand of 4.70%, with the highest rates in Northern Thailand (11.14%) and among HIV-infected individuals (16.33%). Although infection was associated with a two-fold increase in the risk of diarrhea, the association was not statistically significant. Notably, HIV-seropositive individuals had an eight-fold higher risk compared to HIV-seronegative individuals. Future research should expand surveillance in under-represented regions to better understand the epidemiological burden and guide public health interventions.

## 1. Introduction

*Cryptosporidium* is an intracellular protozoan parasite recognized as a significant causative agent of diarrhea worldwide [1]. A relatively small number of *Cryptosporidium* oocysts can establish an infection, leading to outbreaks of cryptosporidiosis, particularly in children and immunocompromised individuals. These outbreaks can arise from the consumption of contaminated food or water, or through direct contact with infected humans or animals [2]. In fact, *Cryptosporidium* was identified as the fifth leading cause of diarrhea in children under five years of age, resulting in over 4.2 million disability-adjusted life years lost and more than 48,000 deaths [3]. A multicenter global study revealed that *Cryptosporidium* infection is the leading cause of deaths from moderate-to-severe diarrhea in children 2–23 months old [4].

*Cryptosporidium* infects gastric and intestinal epithelial cells, causing either self-limiting diarrhea or asymptomatic infection in healthy people; however, in immunocompromised individuals, particularly those with human immunodeficiency virus (HIV)/acquired immunodeficiency syndrome (AIDS), the infection can be particularly severe and life-threatening. In HIV-positive patients, *Cryptosporidium* can lead to chronic diarrhea, which significantly contributes to morbidity and mortality. Studies indicate that individuals with a cluster of differentiation 4 (CD4) count below 200 cells/mm^3^ are at increased risk for severe cryptosporidiosis, often resulting in prolonged gastrointestinal symptoms and complications [5]. The wide-ranging severity of the disease is influenced by factors such as age, nutritional status, and immune competence of the host, as well as the specific *Cryptosporidium* species and genotypes involved [6]. Epidemiological investigations have demonstrated that early-childhood cryptosporidiosis is often linked to malnutrition, growth disorders, and long-term cognitive deficits [7,8,9,10]. Unfortunately, no effective vaccines have been developed to combat this infection [4].

The genus *Cryptosporidium* comprises at least 44 species and over 120 identified genotypes, with *C. hominis* and *C. parvum* accounting for more than 90% of human cryptosporidiosis cases [1]. In Thailand, diverse population dynamics and varying environmental conditions create unique challenges for controlling this infection. However, diagnosis in Thailand remains limited by factors such as inconsistent laboratory capacities, the lack of routine molecular testing, and reliance on conventional microscopy, which may underestimate true prevalence. Although sporadic reports exist, systematic evidence on the prevalence of *Cryptosporidium* and its risk factors remains fragmented, especially across the diverse regions in Thailand [11]. Thus, comprehensive data on *Cryptosporidium* prevalence and its association with diarrhea remain sparse, not to mention the lack of national data to help support policy development. This lack of systematic analyses hinders our understanding of the parasite’s true impact on the Thai population.

This systematic review and meta-analysis aimed to provide a comprehensive assessment of the overall prevalence of *Cryptosporidium* infection in humans across Thailand and evaluate its association with diarrhea, particularly among immunocompromised individuals such as those living with HIV. By synthesizing the available data, this study will offer critical insights into the epidemiological patterns of *Cryptosporidium* infection, which are essential for informing targeted public health strategies and interventions.

## 2. Methods

### 2.1. Registration and Reporting of the Systematic Review

The protocol for this systematic review and meta-analysis was registered in PROSPERO (CRD420251015538). This review’s reporting adhered to the Preferred Reporting Items for Systematic Reviews and Meta-Analyses (PRISMA) guidelines [12].

### 2.2. Systematic Review Framework

The systematic review framework followed the Population, Exposure, Comparison, and Outcomes (PECO) model [13]. P (Population) includes human participants living in Thailand; E (Exposure) is *Cryptosporidium* infections; C (Comparison) is a comparator for each outcome (e.g., non-diarrhea, HIV-negative, HIV-seronegative); and O (Outcome) is the prevalence or odds of *Cryptosporidium* infections in Thailand.

### 2.3. Search Strategy and Databases

A comprehensive literature search was conducted across multiple electronic databases, including PubMed, Embase, Scopus, Journals@Ovid, Nursing & Allied Health Premium, Google Scholar, and the Thai-Journal Citation Index (TCI), from inception to 20 March 2025. The search strategy combined MeSH terms, Emtree terms, and free-text keywords to identify relevant studies on *Cryptosporidium* infections in Thailand. The general search strategy used the following terms: (*Cryptosporidium* OR “*Cryptosporidium parvum*” OR Coccidia OR Cryptosporidiidae OR Cryptosporidiosis) AND (Thailand OR Siam). No restrictions were applied regarding language or publication date. For Google Scholar, the first 200 results were screened, and for the TCI, the keyword “*Cryptosporidium*” was used.

### 2.4. Eligibility Criteria, Article Selection, and Data Extraction

All search results retrieved from the databases were imported into reference management software for deduplication (EndNote version 20.0, Clarivate, Philadelphia, United States). After removing duplicates, titles and abstracts were independently screened by two review authors (MK and AM) based on predefined eligibility criteria. Studies were included if they reported data on the prevalence or associated risk factors of *Cryptosporidium* infection in humans in Thailand. Studies were excluded if they did not involve human subjects (e.g., animal or environmental studies), were conducted outside of Thailand or did not include Thai participants, reported no cases of *Cryptosporidium* infection, or were review articles, conference abstracts, editorials, or case reports.

For the meta-analyses, studies were further selected based on their specific outcomes of interest. Cross-sectional or observational studies that reported sufficient data to calculate the prevalence of *Cryptosporidium* infection (i.e., number of cases and total sample size) were included in the pooled prevalence analysis. For the analysis of the association between *Cryptosporidium* infection and HIV status, only studies that reported infection data separately for HIV-seropositive and HIV-seronegative individuals were included to estimate the odds ratio of infection. Similarly, studies that provided data on *Cryptosporidium* infection in individuals with and without diarrhea were selected for the analysis assessing the association between infection and diarrheal disease. As not all studies reported data relevant to each of these outcomes, the number of studies included in each analysis varied accordingly.

From each eligible study, relevant data were independently extracted by two review authors (MK and AM) using a standardized data extraction form. The extracted information included the following: first author and year of publication; study design; study location and the corresponding region of Thailand (e.g., North, Central, South, Northeast); and the year(s) during which the study was conducted. Characteristics of the study population, including types of participants, clinical signs or symptoms reported, mean age (mean ± SD or median ± range), age range, and percentage of male participants, were recorded. Study sample size (number of participants) and the number of detected *Cryptosporidium* infections were documented. Additionally, information on the diagnostic methods used for *Cryptosporidium* detection, including both general methods (e.g., microscopy, PCR) and specific detection techniques, as well as on any genes targeted in molecular assays, was extracted. Any disagreements during the selection and data extraction processes were resolved by discussion or consultation with another review author (JJ). A final list of the included studies was compiled for quality assessment.

### 2.5. Methodological Quality Assessment

The Joanna Briggs Institute (JBI) critical appraisal tools were used to assess the methodological quality and risk of bias in different types of observational studies [14]. For cross-sectional studies, a checklist was used to evaluate aspects such as the clarity of the inclusion criteria, the validity and reliability of the measurement tools, and whether potential confounding factors were identified and addressed. A case–control checklist focused on ensuring that cases and controls were appropriately matched or comparable, that exposures were measured consistently and accurately, and that important confounders were considered. For cohort studies, an appraisal emphasized whether groups were similar at baseline, if exposures were measured in a valid and reliable manner, and whether outcomes were assessed using objective criteria over an adequate follow-up period.

### 2.6. Data Syntheses

Data from the cross-sectional or observational studies were synthesized by meta-analysis to estimate the pooled prevalence of *Cryptosporidium* infections and to assess their associations with diarrhea and HIV status in Thailand. A random-effects model was employed for the primary analysis, which was chosen to account for expected between-study heterogeneity (e.g., differences in populations, study designs, and diagnostic methods) by assuming that the true effect size varied across the included studies. The restricted maximum likelihood (REML) estimator was used for the between-study variance (τ^2^) [15]. Prevalence data were pooled using a logit transformation to ensure accurate estimation for proportions, and Clopper–Pearson confidence intervals were applied. For effect size synthesis in association studies, odds ratios (ORs) with 95% confidence intervals (CIs) were calculated using the inverse variance method. Separate meta-analyses were conducted to assess the association between *Cryptosporidium* infections and (1) diarrhea and (2) HIV serostatus (seropositive vs. seronegative). To explore sources of heterogeneity, meta-regression and subgroup analyses were conducted. Subgroup analyses were specifically designed to investigate whether effect estimates varied across predefined study characteristics, including study design (e.g., cross-sectional or observational), geographical regions of Thailand, participant demographics, and diagnostic methods. The inclusion of these specific categories was based on the a priori hypothesis that these factors could be significant moderators of the pooled effect size. Heterogeneity was quantified using *I*^2^, τ^2^, and Q statistics. Sensitivity analyses were conducted to assess the robustness of the findings. This involved the comparison of results from the primary random-effects model with those from a fixed-effects model. The fixed-effects model assumes a single true effect size across all studies and does not account for heterogeneity. To assess the robustness of the findings, sensitivity analyses were performed. These involved comparing the results of the primary random-effects model with those from a fixed-effects model, conducting a ‘leave-one-out’ analysis to identify influential studies and outliers, and rerunning the meta-analysis after excluding studies with a high risk of bias. A continuity correction of 0.5 was applied to studies with zero events. Publication bias was evaluated using funnel plots and Egger’s test, limited to analyses that included ≥10 studies [16,17]. All analyses were conducted using RStudio (Version 2024.04.2+764) [18], and forest plots were generated to visualize the pooled estimates.

## 3. Results

### 3.1. Search Results

**Figure 1:**
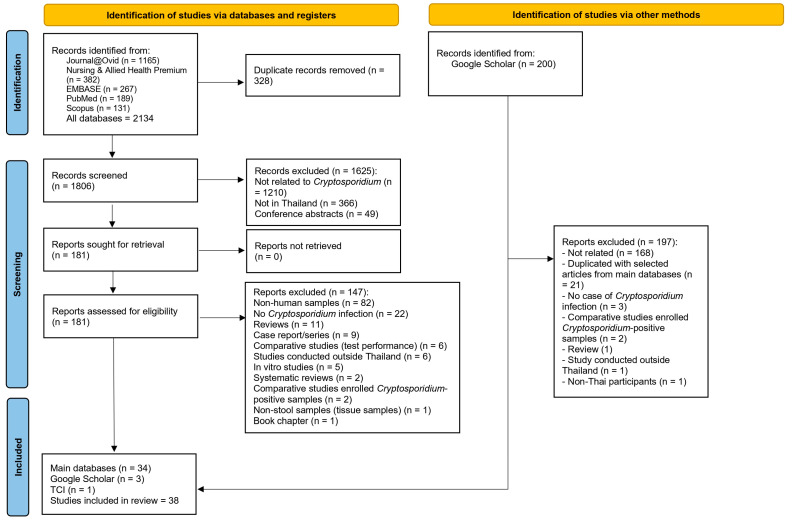
PRISMA 2020 flow diagram illustrating the study selection process for the systematic review on *Cryptosporidium* infections in Thailand.

A total of 2134 records were initially identified from five databases: Journals@Ovid (1165), Nursing & Allied Health Premium (382), EMBASE (267), PubMed (189), and Scopus (131). After removing 328 duplicate records, 1806 records were screened. Of these, 1625 were excluded for reasons such as not being related to *Cryptosporidium* (1210), having not been conducted in Thailand (366), or being conference abstracts (49). A total of 181 full-text reports were assessed for eligibility, and 147 were excluded due to issues like non-human samples (82), lack of *Cryptosporidium* infection data (22), or being reviews, case reports, or irrelevant comparative studies, or for other reasons (43). An additional search via Google Scholar (200 records) contributed 3 more studies after the exclusion of 197 studies, while 1 study was retrieved using the Thai Citation Index (TCI). In total, 38 studies were included in the final review [11,19,20,21,22,23,24,25,26,27,28,29,30,31,32,33,34,35,36,37,38,39,40,41,42,43,44,45,46,47,48,49,50,51,52,53,54,55]—34 from the main databases, 3 from Google Scholar, and 1 from TCI (Figure 1).

### 3.2. Characteristics of Included Studies

A total of 38 studies with 24,759 participants were included. The majority of the studies were conducted between 2000 and 2022 (n = 25; 65.8%) and were cross-sectional, prospective, or retrospective observational studies (n = 35; 92.1%; Table 1). Most studies were conducted in Central Thailand (n = 28; 73.7%), with Bangkok being the most common specific location (n = 18; 47.4%), followed by other provinces such as Nonthaburi and Lop Buri. A few studies were conducted in other regions, including the west (e.g., Ratchaburi), north (e.g., Phayao), south (e.g., Songkhla), and northeast (e.g., Khon Kaen), with some covering multiple areas or border regions. Regarding age groups, participants were children (n = 11; 29.0%), adults (n = 9; 23.7%), mixed-age groups (n = 9; 23.7%), or not specified (n = 9; 23.7%). The most common participant groups were HIV-related groups (n = 23; 60.5%) and child subgroups (n = 8; 21.1%). For *Cryptosporidium* detection, most studies (n = 32; 84.21%) used standard methods, such as acid-fast staining, enzyme immunoassays, immunofluorescence assays, or PCR techniques, while a few used non-standard methods (n = 2; 5.26%) or did not specify the method used (n = 4; 10.53%). Among the 32 studies that used standard methods to identify *Cryptosporidium*, 6 (18.8%) employed PCR techniques targeting the SSU rRNA gene.

### 3.3. Risk of Bias

For cross-sectional and observational studies, the majority of the studies were of low risk for bias (86.8%, Table S3). Three studies (7.9%) were classified as having a high risk of bias related to confounding factors, measurement reliability, and analytical clarity. One study (2.6%) was rated as having a moderate risk of bias, with 62.5% of the criteria met. For the case–control studies, all three studies were rated as having a low risk of bias, indicating good methodological quality overall.

### 3.4. Prevalence of Cryptosporidium Infections in Thailand

**Figure 2:**
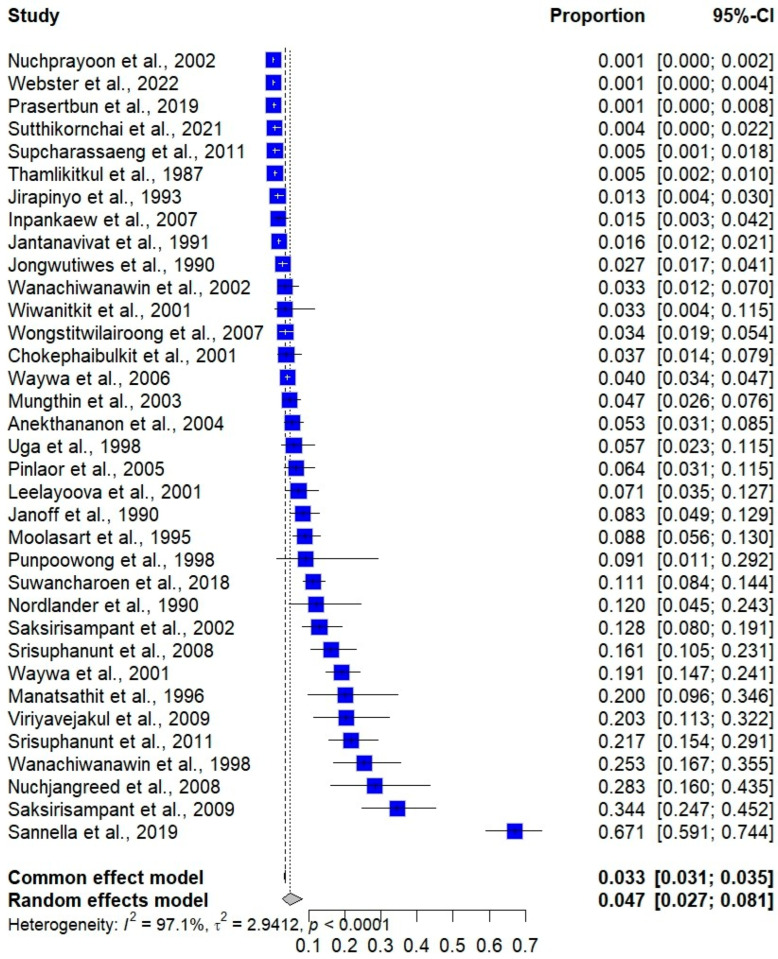
Forest plot showing the prevalence of *Cryptosporidium* infections in individual studies conducted in Thailand, along with pooled estimates. Each row represents a study, with the blue squares indicating the point estimates of prevalence and the horizontal lines representing the 95% confidence intervals (CIs). The size of each square reflects the weight of the study in the meta-analysis. The diamond at the bottom represents the pooled prevalence estimate under the common-effects model and under the random-effects model [11,19,22,23,24,25,26,27,28,29,30,31,32,33,34,35,36,37,38,39,40,41,42,43,44,45,46,47,48,49,51,52,53,54,55].

**Figure 3:**
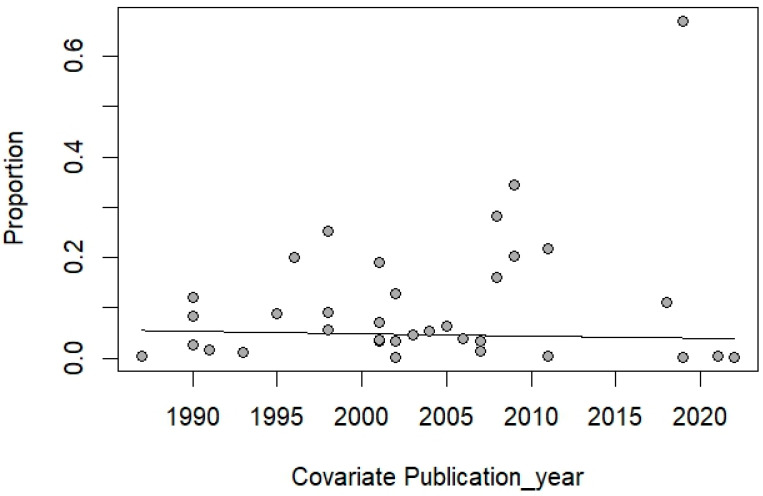
The scatter plot shows the trend in *Cryptosporidium* infection prevalence over time by publication year. Each point represents the proportion reported in an individual study, with the x-axis indicating the year of publication and the y-axis representing the reported prevalence. The fitted line indicates no clear upward or downward trend over time.

The meta-analysis, which included 35 studies (cross-sectional or observational studies) with a total of 23,824 participants and 780 confirmed cases, estimated a pooled prevalence of 4.70% (95% CI: 2.68% to 8.13%) using the random-effects model, which accounted for between-study variability (*I*^2^: 97.1%, Figure 2). There was no significant alteration in the trend in *Cryptosporidium* infection prevalence over time from 1987 to 2022 (Figure 3). This lack of a significant trend was statistically supported by a meta-regression analysis with publication year as a covariate (*p* = 0.7526), suggesting no statistically significant increase or decrease in prevalence over the study period.

The meta-regression analysis explored the influence of various covariates on the pooled prevalence of *Cryptosporidium* infections in Thailand (Table 2, Table S4). The analysis showed significant residual heterogeneity (*p* < 0.001) across all covariates, with high *I*^2^ values indicating a substantial variability that could not be fully explained by the tested moderators. Among the covariates, age groups (*p* = 0.0006), types of participants (*p* < 0.001), male percentage (*p* = 0.0069), and diarrheal status (*p* < 0.001) were significant predictors of prevalence.

The subgroup analysis further illustrates how the prevalence of *Cryptosporidium* infection varied across different categories (Table S4). In studies with a cross-sectional design, the pooled prevalence was 4.59% (95% CI: 2.27–9.06; *I*^2^ = 96.7%; 24 studies). Prospective and retrospective observational studies reported pooled prevalences of 5.05% (six studies) and 4.68% (five studies), respectively. Regionally, the highest pooled prevalence was observed in Northern Thailand (11.14%, one study), while the lowest was in Western Thailand (0.51%, three studies). These differences were statistically significant (*p* < 0.0001), reflecting geographic heterogeneity. Age was another significant factor (*p* = 0.0014), with adults showing the highest prevalence (18.27%, nine studies), followed by children (3.19%, nine studies), and mixed-age groups (1.43%, nine studies). Participant types also significantly influenced prevalence (*p* < 0.0001); HIV-infected individuals had a markedly high prevalence (16.33%, 16 studies), while the lowest was seen among villagers and schoolchildren (0.14%, 1 study). Diarrheal status was a strong predictor (*p* = 0.0002), with those having diarrhea alone (7.94%, 10 studies) or both diarrheal and non-diarrheal symptoms (7.19%, 17 studies) showing significantly higher prevalence compared to non-diarrheal participants (0.14%, 1 study). The detection method did not result in significant subgroup differences (*p* = 0.5463). The pooled prevalence of *Cryptosporidium* detected using PCR and non-PCR methods was comparable, at 5.99% (6 studies) and 5.41% (33 studies), respectively. At the regional and provincial levels, evidence of infections was mostly reported in Central Thailand, where provinces like Lop Buri and Bangkok/Nonthaburi combined reported extremely high prevalence estimates of 32.35% (two studies) and 29.54% (four studies), respectively, while Bangkok alone had a much lower estimate (2.86%, 17 studies).

Among the studies using PCR techniques, the identified species included *C. parvum* [33,36,40,41], *C. hominis* [40,41,42], *C. meleagridis* [40,41,42], *C. canis* [40,41], *C. felis* [40,41,44], and *C. suis* [40]. The meta-analysis further detailed the pooled prevalence of these species: *C. hominis* (21.07%, three studies), *C. meleagridis* (5.17%, three studies), *C. canis* (2.64%, two studies), *C. parvum* (2.02%, four studies), *C. felis* (1.16%, three studies), and *C. suis* (3.87%, one study) (Table 3).

### 3.5. Risk of Diarrhea in Participants with Cryptosporidium Infections

The meta-analysis examining the association between *Cryptosporidium* infections and the odds of having diarrhea, based on eight studies, found that although individuals with the infection appeared to have higher odds of diarrhea, this association was not statistically significant (*p* = 0.3969; OR: 2.00; 95% CI: 0.67–5.99; *I*^2^: 70%; eight studies; number of participants: 2820; Figure 4).

The subgroup analysis showed a significant increase in odds in studies conducted in Central (OR = 3.71; five studies) and Northeastern Thailand (OR = 5.07; one study), whereas studies conducted in Western Thailand showed a significantly decreased risk (OR = 0.37; two studies, Table S5). At the provincial level, the highest odds were reported in studies conducted in Nonthaburi (OR = 82.1; one study) and Khon Kaen (OR = 5.07; one study). Studies that enrolled children showed no significant association (OR = 0.89; four studies), while those involving children with diarrhea reported markedly higher odds (OR = 22.3; two studies). Studies that enrolled pre-school children showed lower odds of diarrhea (OR = 0.37; two studies). Among HIV-infected individuals, a moderate but non-significant increase in odds was observed (OR = 2.50; two studies).

### 3.6. Association Between Cryptosporidium Infections and HIV Infection Status

The meta-analysis examining the association between *Cryptosporidium* infections and HIV infections based on the random-effects model showed no significant association (*p*: 0.19; 95% CI: 0.22–1497.8; *I*^2^: 90.6%; three studies; number of participants: 4550; Figure 5).

The meta-analysis examining the association between *Cryptosporidium* infection and HIV serostatus using the random-effects model showed that HIV-seropositive individuals had significantly higher odds of *Cryptosporidium* infection (*p*: 0.0062; OR: 8.15; 95% CI: 1.82–36.50; *I*^2^: 0%; two studies; number of participants: 278; Figure 6).

### 3.7. Sensitivity Analysis

**Figure 4:**
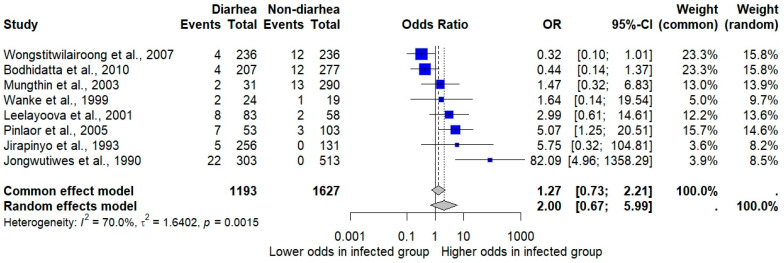
Forest plot showing the association between *Cryptosporidium* infections and the odds of having diarrhea. The plot displays the odds ratios (ORs) and 95% confidence intervals (CIs) from eight individual studies. The size of each square reflects the weight of the study in the analysis, and horizontal lines represent 95% CIs. The pooled ORs using both common- and random-effects models are shown at the bottom [21,26,27,28,31,35,50,55].

A sensitivity analysis was conducted to examine the difference in results when using a different statistical model for pooling the data. The meta-analysis examining the pooled prevalence of *Cryptosporidium* infections in Thailand using a fixed-effect model demonstrated a pooled prevalence of 3.27% (95% CI: 3.06% to 3.51%, Figure 2). The meta-analysis examining the association between *Cryptosporidium* infections and the odds of having diarrhea, based on the fixed-effects model, also showed a non-significant association between *Cryptosporidium* infections and the odds of having diarrhea (*p* = 0.3969; OR = 1.27; 95% CI: 0.73–2.21; Figure 4). The meta-analysis examining the association between *Cryptosporidium* infections and HIV infections (infected vs. uninfected) based on the fixed-effects model showed a statistically significant association (*p*: 0.003; 95% CI: 1.83–19.32; *I*^2^: 90.6%; three studies; number of participants: 4550; Figure 5). The meta-analysis examining the association between *Cryptosporidium* infections and HIV serostatus (seropositive vs. seronegative) based on the fixed-effects model showed similar results with the random-effects model (*p*: 0.0062; 95% CI: 1.82–36.50; *I*^2^: 0%; two studies; number of participants: 278; Figure 6).

A sensitivity analysis showed that when high-risk-of-bias studies were excluded and the meta-analysis was rerun using the random-effects model, the pooled prevalence was 4.57% (95% CI: 2.68% to 8.13%), which was slightly lower than the overall pooled prevalence of 4.70%.

A leave-one-out analysis suggested that no single study was an extreme outlier or had a disproportionately large influence on the overall pooled prevalence estimate (Table S6). The heterogeneity remained high and stable, suggesting that the high level of heterogeneity was not caused by one or two outlying studies.

### 3.8. Publication Bias

The funnel plot appeared symmetrical around the pooled estimate, suggesting no strong indication of publication bias. This visual assessment aligns with the results of the Egger’s test, which showed no statistically significant asymmetry (*p* = 0.5206). While a few studies lay outside the funnel boundaries—particularly those with high standard errors and extreme effect sizes—this pattern is more likely due to the substantial heterogeneity among the studies (*I*^2^ = 97.1%) rather than publication bias. The publication bias of the meta-analysis examining the association between *Cryptosporidium* infection and the odds of having diarrhea could not be assessed because the number of studies incorporated in the meta-analysis was less than 10.

## 4. Discussion

This systematic review and meta-analysis estimated the overall pooled prevalence of *Cryptosporidium* infection in Thailand at 4.70%, which is lower than the global prevalence of 7.6% reported in a previous meta-analysis [56]. That global study also indicated higher prevalence rates among participants under five years of age, those residing in low-income countries, and individuals with gastrointestinal symptoms [56]. In a previous meta-analysis, which included three studies from Thailand, the reported prevalence of *Cryptosporidium* infection was 5.7% among patients and 1.9% among schoolchildren [56]. The findings of the present study are consistent with these results, showing a similarly low prevalence among schoolchildren in Thailand (0.39% vs. 1.9%). However, both figures are markedly lower than the global prevalence reported in that study, where schoolchildren had the highest prevalence at 16.4% [56]. This discrepancy may reflect improvements in sanitation, hygiene, water treatment infrastructure, and public health interventions in Thailand.

The substantial heterogeneity observed in the meta-analysis (*I*^2^ = 97.1% for pooled prevalence) suggests substantial heterogeneity among the included studies. This high heterogeneity was likely caused by a combination of epidemiological, methodological, and population-related factors across the diverse studies conducted in Thailand. For epidemiological contributors, the highest prevalence rates were observed in Northern Thailand (11.14%) and Northeastern Thailand (6.41%), although these estimates were based on single studies in each region. The majority of the studies (27 studies) were conducted in Central Thailand, where the overall prevalence was also relatively high at 6.08%, indicating a concentrated research focus in this area, particularly in Bangkok (17 studies). However, the prevalence of infection in Bangkok itself was lower, at 2.86%, compared to other provinces in Central Thailand, such as Lop Buri (32.35%, two studies) [33,39] and Nonthaburi (8.57%, four studies) [27,29,30,38]. Interestingly, studies conducted across both Bangkok and Nonthaburi reported a high combined prevalence of 29.54% (four studies) [40,42,49,51]. These striking regional differences are most likely driven by the specific study populations sampled. The exceptionally high prevalence rates in Lop Buri and Nonthaburi can be directly attributed to the presence of major national centers for HIV/AIDS care: Wat Phrabat Namphu in Lop Buri and the Bamrasnaradura Infectious Diseases Institute in Nonthaburi. Research conducted in these provinces would logically focus on these high-risk, immunocompromised patient groups, who are significantly more susceptible to opportunistic infections like cryptosporidiosis. This targeted sampling explains the inflated prevalence figures compared to those from Bangkok, which likely included a broader, more general population. While this sampling bias appears to be the primary explanation, contributing environmental and socio-demographic factors should not be overlooked. The rapid urbanization in provinces like Nonthaburi, for example, can strain existing water and sanitation infrastructure, potentially increasing the risk of transmission for waterborne pathogens. This issue is compounded by a nationwide concern over water quality. Although national data indicate over 90% of Thais have access to improved water sources, studies have found that as little as 40% of household water is suitable for consumption, i.e., potable [57]. This suggests that even with widespread access to facilities, the quality of water and sanitation services may be a key factor in the regional prevalence of *Cryptosporidium* infections.

For methodological contributors, the choice of detection method did not yield significant differences in prevalence rates (*p* = 0.5463), with comparable rates recorded for PCR (5.99%) and non-PCR methods (5.41%). While this suggests both methodologies are effective, the inherent differences in their sensitivity and specificity can still contribute to heterogeneity, even if not statistically significant in the subgroup analysis. PCR methods are generally more sensitive and specific than microscopy or enzyme immunoassays, potentially leading to higher detection rates and differing prevalence estimates across studies utilizing different techniques. The molecular data from this analysis reveal that anthroponotic (human-to-human) transmission is the main driver of infection in Thailand. This is evidenced by the clear predominance of *Cryptosporidium hominis*, with a pooled prevalence of 21.07% in the included studies [40,41,42]. This transmission pathway is often facilitated by contaminated water systems or direct person-to-person contact, particularly in densely populated areas [58]. Despite this, the role of zoonotic transmission is significant and diverse. The high prevalence of *C. meleagridis* (5.17%), a species typically found in birds, strongly suggests an environmental contamination route by avian hosts living in close proximity to humans, which is supported by previous Thai studies that identified this parasite in domestic pigeons and water [59,60]. This directly links the species to environmental contamination and specific animal hosts that live in close proximity to humans. Furthermore, the presence of *C. canis*, *C. felis*, and *C. suis* implicates dogs, cats, and pigs as important reservoirs [59,61], highlighting transmission risks from both domestic and agricultural animals. Interestingly, *C. parvum*, a major zoonotic species linked to cattle globally, was found at a relatively low prevalence of 2.02%. This could reflect a true lower prevalence in local livestock or a sampling bias in the original studies away from rural, cattle-farming communities. This finding suggests that unlike in many Western countries, cattle may play a less dominant role in the zoonotic transmission of cryptosporidiosis in Thailand. Taken together, these findings support the need for a “One Health” approach to effectively control *Cryptosporidium*, addressing human sanitation, agricultural practices, and the health of domestic animals concurrently. Future large-scale molecular studies across varied geographic and demographic settings are essential to further refine our understanding of these complex transmission dynamics.

In Thailand, the meta-analysis showed the highest prevalence of *Cryptosporidium* infections among HIV-infected individuals (16.33%, 16 studies), consistent with evidence that infections are more severe and persistent in immunocompromised hosts [62,63]. This aligns with a previous meta-analysis reporting a prevalence of 11.2% in HIV-infected individuals [64]. Nevertheless, that meta-analysis found no significant association between *Cryptosporidium* infection and HIV infection (*p* = 0.19). This comparison, based on only three studies, yielded an extremely wide confidence interval (OR 0.22–1497.8), indicating substantial imprecision and rendering the estimate statistically unstable and difficult to interpret. For HIV serostatus, the present study found a lower pooled prevalence of 6.12% among studies that included both HIV-seropositive and -seronegative patients. This finding suggests that HIV serostatus may influence the risk of *Cryptosporidium* infection. Furthermore, this meta-analysis showed that HIV-seropositive individuals had an 8.15-fold higher risk of *Cryptosporidium* infection compared to HIV-seronegative individuals, highlighting the parasite’s opportunistic behavior in immunocompromised hosts. The susceptibility of AIDS patients to *Cryptosporidium* infection can be explained by the immune response against the infection. CD4+ cells are immune cells that respond to gastrointestinal pathogens [65]. Individuals with low CD4 counts are at risk for *Cryptosporidium* infection due to immunosuppression [64]. In addition, individuals with CD4 counts below 200 cells/mm^3^ are at greater risk for severe and persistent cryptosporidiosis [66].

The present meta-analysis showed that the pooled prevalence of *Cryptosporidium* infections among diarrheic patients only, both diarrheic and non-diarrheic, and non-diarrheic only was 7.94%, 7.19%, and 0.14%, respectively. This meta-analysis found a pooled OR of 2.00 for the association between *Cryptosporidium* infection and diarrhea; however, this was not statistically significant (*p* = 0.3969), and its high heterogeneity (*I*^2^ = 70%) suggests caution in interpretation. While certain subgroup analyses showed significant associations in specific settings, such as studies conducted in Central and Northeastern Thailand or among children with diarrhea, these findings were often based on very few studies. Therefore, the current evidence is insufficient to draw firm conclusions regarding a causal relationship between *Cryptosporidium* infection and diarrhea in Thailand, and larger, well-designed studies are warranted to clarify this association.

### 4.1. Study Limitations

Several limitations of this systematic review and meta-analysis should be acknowledged. Many of the subgroup findings were based on a small number of studies, which limits the generalizability of these results. Data from certain areas, especially Southern and Northeastern Thailand, were extremely limited, in some cases relying on a single study. This uneven geographical coverage suggests that observed regional differences may be influenced by sampling bias and may not fully represent the true distribution of *Cryptosporidium* infection across Thailand. The current epidemiological map is therefore incomplete and should be refined through further research in under-represented regions. The high heterogeneity observed in the main analysis (*I*^2^ = 97.1%) indicates substantial variability among the included studies. Although meta-regression was conducted, it did not fully account for all the sources of heterogeneity. This unexplained heterogeneity may be due to unmeasured factors, such as seasonal variation, as data on the season of data collection were not consistently available across the included studies. Climatic conditions, such as seasonal flooding or variations in rainfall, could also influence the regional epidemiology of *Cryptosporidium* species [67,68]. As such, the pooled prevalence estimate should be interpreted with extreme caution, as it may be subject to substantial error due to unexplained between-study variability.

Furthermore, variation in diagnostic methods, including the use of non-standard techniques, may have led to under- or overestimation in certain studies. PCR is inherently more sensitive than microscopy and immunoassays, which could lead to higher detection rates in some settings. Therefore, direct comparisons between prevalence estimates from different diagnostic techniques should be interpreted with caution. The association analyses, particularly those involving HIV status and HIV serostatus, were limited by the small number of eligible studies included in the meta-analysis, which were three and two studies, respectively. Although formal tests for publication bias (e.g., Egger’s test) did not indicate significant asymmetry, this assessment was only possible for outcomes with ≥10 studies. For analyses with fewer than 10 studies, the possibility of publication bias could not be entirely ruled out. Furthermore, potential confounding factors were not consistently accounted for across the included studies, which may have influenced the reported prevalence estimates and associations. Therefore, the findings should be interpreted with caution.

### 4.2. Implications

One critical challenge in understanding the true prevalence of *Cryptosporidium* infection is the limited incorporation of specific diagnostic methods into routine clinical practice. In Thailand, routine stool examinations typically do not include modified acid-fast staining, which is the standard technique for detecting *Cryptosporidium* oocysts. This means case detection often depends on research studies or targeted testing, rather than ongoing clinical surveillance. While integrating modified acid-fast staining into standard stool examinations, particularly for patients presenting with diarrhea or individuals at high risk (e.g., people living with HIV), could substantially enhance surveillance capacity and enable more effective public health responses, this approach would require additional expenses for reagents, equipment maintenance, and staff training. Its feasibility should be supported by a cost effectiveness analysis comparing the public health benefits of improved detection with its financial and logistical demands. In the absence of such an analysis, a more pragmatic initial step could involve the targeted use of modified acid-fast staining in high-risk groups (e.g., immunocompromised patients, outbreak investigations, and regions with known higher prevalence). This targeted approach could enhance surveillance capacity while minimizing costs, and also generate data to inform future nationwide policy decisions. Furthermore, the under-representation of certain Thai regions, with limited data from Southern and Northeastern Thailand, may have caused the pooled national figure to be either over- or underestimated. This highlights the need for more targeted research in these areas to provide a more accurate and representative picture of the parasite’s distribution in the country.

The presence of multiple *Cryptosporidium* species, particularly *C. hominis*, *C. parvum*, and *C. meleagridis*, has significant public health implications for Thailand. The predominance of *C. hominis* suggests that waterborne transmission through contaminated water is a primary concern, highlighting the need for improved sanitation and water treatment. In contrast, the presence of *C. parvum*, a zoonotic species often linked to livestock [69], means surveillance and control measures must extend beyond human populations to include animal and environmental health. Finally, the broad host range of *C. meleagridis* points to potential for both human and animal transmission [70]. By understanding the specific species circulating in Thailand, public health interventions can be more effectively implemented, targeting not only water safety but also hygiene education for at-risk groups.

## 5. Conclusions

This systematic review and meta-analysis estimated the pooled prevalence of *Cryptosporidium* infection in Thailand at 4.70%, with the highest prevalence observed in Northern Thailand (11.14%) and among HIV-infected individuals (16.33%). Although *Cryptosporidium* infection was associated with a twofold increase in the risk of diarrhea, this association was not statistically significant. In contrast, HIV-seropositive individuals had an approximately eightfold higher risk of infection compared to HIV-seronegative individuals, highlighting a critical vulnerability in immunocompromised populations. To improve early detection and more accurately monitor the burden of cryptosporidiosis, integrating routine diagnostic testing, such as modified acid-fast staining or molecular methods, into national surveillance protocols is strongly recommended. Including *Cryptosporidium* testing in routine stool examinations, particularly for patients presenting with diarrhea and individuals at high risk (e.g., people living with HIV), would substantially enhance surveillance capacity and enable more effective public health responses. Future research should prioritize addressing these regional data gaps, especially in Southern and Northeastern Thailand, by expanding surveillance to under-represented areas and populations, using standardized diagnostic methods to provide a more comprehensive and reliable national epidemiological picture.

## Figures and Tables

**Figure 5:**
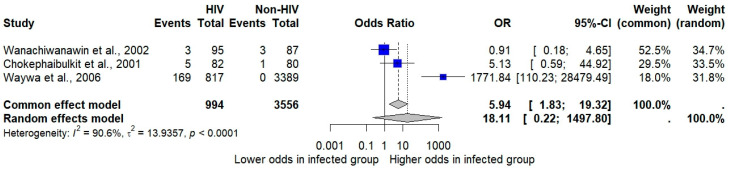
Forest plot showing the association between *Cryptosporidium* infections and HIV status. Blue squares represent the odds ratios (ORs) for individual studies, with the size of each square proportional to the study’s weight in the analysis. Horizontal lines indicate 95% confidence intervals (CIs), and the vertical dashed line at OR = 1 represents the line of no effect. The diamond at the bottom shows the pooled OR estimate from the random-effects model [22,48,51].

**Figure 6:**
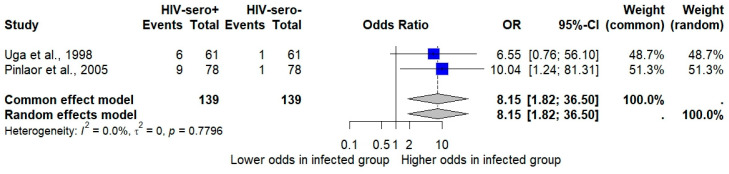
Forest plot showing the association between *Cryptosporidium* infections and HIV serostatus. Blue squares represent the odds ratios (ORs) for each study, with horizontal lines indicating 95% confidence intervals (CIs), and the diamond represents the pooled OR from both common and random-effects models [35,46].

**Table 1:** Characteristics of included studies (n = 38).

Characteristics	Number of Studies (*n*)	%
**Publication years**		
Before 2000	13	34.2
After 2000	25	65.8
**Study designs**		
Cross-sectional, prospective, or retrospective observational studies	35	92.1
Case–control study	3	7.89
**Areas of Thailand**		
Central Thailand	28	73.7
- Bangkok	18	47.4
- Nonthaburi	4	10.5
- Bangkok, Nonthaburi	4	10.5
- Lop Buri	2	5.26
Western Thailand	4	10.5
- Kanchanaburi	2	5.26
- Ratchaburi	1	2.63
- Thailand–Burma border	1	2.63
Southern Thailand (Songkhla)	1	2.63
Northern Thailand (Phayao)	1	2.63
Northeastern Thailand (Khon Kaen)	1	2.63
Thai–Cambodian border (not specified province)	2	5.26
Multi-areas (Chiang rai, Nan, Tak, Ratchaburi, Loei, Chumphon, Sa Kaeo)	1	2.63
**Age groups of participants**		
Children	11	28.9
Adults	9	23.7
Mixed-age groups	9	23.7
Not specified	9	23.7
**Participants**		
HIV-related groups	23	60.5
Child subgroups	8	21.1
Participants suspected of the parasite	3	7.89
Monks or nuns	1	2.63
Adult patients	1	2.63
Refugees	1	2.63
Villagers and school children	1	2.63
**Detection method for *Cryptosporidium***		
Standard method	32	84.2
- PCR method	6	15.8
- Non-molecular methods *	26	81.2
Non-standard method	2	5.26
Not specified	4	10.5

Abbreviations: HIV, Human Immunodeficiency Virus. * Acid-fast stain, modified acid-fast stain, modified Ziehl–Neelsen stain, enzyme immunoassay, immunofluorescence assay, modified dimethylsulfoxide method.

**Table 2:** Subgroup analysis of the pooled prevalence of *Cryptosporidium* infections in Thailand.

Covariate	Subgroup	Pooled Prevalence (%) [95% CI]	*I*^2^ (%)	Number of Studies
Overall prevalence		4.70 [0.03; 0.08]	97.1	35
Publication year	Before 2000	5.06 [2.47; 10.09]	95.4	11
	2000–2022	4.47 [2.08; 9.34]	97.3	24
Study design	Cross-sectional study	4.59 [2.27; 9.06]	96.7	24
	Prospective observational study	5.05 [2.61; 9.54]	82.4	6
	Retrospective observational study	4.68 [0.67; 26.32]	98.9	5
Regions of Thailand	Central Thailand	6.08 [3.36; 10.8]	97.6	27
	Western Thailand	0.51 [0.08; 3.26]	91.8	3
	Northern Thailand	11.1 [8.56; 14.4]	N/A	1
	Northeastern Thailand	6.41 [3.48; 11.5]	N/A	1
	Southern Thailand	5.74 [2.76; 11.6]	N/A	1
	Northeastern, Southern, and Eastern Thailand	0.14 [0.02; 1.01]	N/A	1
	Not specified	12.00 [5.49; 24.24]	N/A	1
Age group of participants	Children	3.19 [1.80; 5.60]	87.9	9
	Adults	18.27 [8.05; 36.3]	96.1	9
	Mixed-age groups	1.43 [0.40; 4.90]	95.4	9
	Not specified	5.47 [2.40; 12.0]	95.3	8
Participants	HIV-infected patients	16.33 [10.93; 23.69]	94.4	16
	HIV-infected and uninfected patients	4.02 [3.51; 4.61]	0.0	4
	Participants suspected of parasite infections	0.44 [0.11; 1.66]	95.8	3
	Children with diarrhea	3.27 [1.18; 8.73]	87.1	3
	HIV-seropositive and -seronegative patients	6.12 [3.83; 9.62]	0.0	2
	Monks or nuns	1.47 [0.48; 4.46]	N/A	1
	Orphanage children	8.29 [5.22; 12.93]	N/A	1
	Villagers and school children	0.14 [0.02; 1.01]	N/A	1
	Adult patients	0.51 [0.13; 2.03]	N/A	1
	School children	0.39 [0.06; 2.74]	N/A	1
	Pre-school children	3.39 [2.09; 5.46]	N/A	1
	Refugees	0.11 [0.03; 0.43]	N/A	1
Detection method for *Cryptosporidium*	Standard method	4.61 [2.41; 8.64]	97.5	29
	- PCR method	5.99 [0.71; 36.3]	96.5	6
	- Non-PCR methods	5.41 [3.18; 9.05]	97.0	23
	Non-standard method	10.56 [2.21; 38.16]	89.0	2
	Not specified	3.82 [1.29; 10.77]	80.7	4
Diarrheal status	Diarrheic	7.94 [4.11; 14.79]	85.8	10
	Diarrheic and non-diarrheic	7.19 [3.70; 13.48]	97.9	17
	Non-diarrheic	0.14 [0.02; 1.01]	N/A	1
	Not specified	1.16 [0.25; 5.22]	97.2	7

N/A, not assessed.

**Table 3:** Prevalence of *Cryptosporidium* species in Thailand (*n* = 1394).

Cryptosporidium Species	n	Pooled Prevalence (%)	95% CI (%)	I^2^ (%)	Number of Studies
*C. parvum*	20	2.02	0.46; 8.49	81.9	4
*C. hominis*	85	21.07	12.31; 33.68	87.8	3
*C. meleagridis*	27	5.17	1.95; 13.02	83.9	3
*C. canis*	13	2.64	0.43; 14.64	83.0	2
*C. felis*	9	1.16	0.27; 4.80	74.1	3
*C. suis*	6	3.87	1.75; 8.35	N/A	1

N/A, not assessed.

## Data Availability

All data relating to the present study are available in this manuscript, Tables S1–S6.

## Supplementary Materials

The following supporting information can be downloaded at https://www.mdpi.com/article/10.3390/medsci13030156/s1, Table S1: Search terms; Table S2: Details of included studies; Table S3: Risk of bias; Table S4: Meta-regression and subgroup analysis of the prevalence of *Cryptosporidium* infection in Thailand; Table S5: Meta-regression and subgroup analysis of the risk of *Cryptosporidium* infection and diarrhea; Table S6: Influential analysis.
