# Supplementary material for: Prevalence of Cryptosporidium Infections in Thailand and Its Association with HIV and Diarrhea: A Systematic Review and Meta-Analysis
Source: Med Sci (Basel). 2025 Aug 26;13(3):156. doi: 10.3390/medsci13030156 (PMC12452679; doi:10.3390/medsci13030156)
Supplement: Supplementary file 1 [file medsci-13-00156-s001.zip › Table S1. Search terms.pdf]

Table S1. Search terms

General keywords

(*Cryptosporidium* OR "*Cryptosporidium parvum*" OR Coccidia OR Cryptosporidiidae OR Cryptosporidiosis) AND (Thailand OR Siam)

PubMed 19 March 2025

No.	Key concept	Search terms	Results
1.	*Cryptosporidium*	*Cryptosporidium*[MeSH Terms] OR "*Cryptosporidium parvum*"[MeSH Terms] OR Coccidia[MeSH Terms] OR Cryptosporidiidae[MeSH Terms] OR Cryptosporidiosis[MeSH Terms] OR *Cryptosporidium*[Text Word] OR "*Cryptosporidium parvum*"[Text Word] OR Coccidia[Text Word] OR Cryptosporidiidae[Text Word] OR Cryptosporidiosis[Text Word]	39,947
2.	Thailand	Thailand[Text Word] OR Thailand[MeSH Terms] OR Siam[Text Word] OR Siam[MeSH Terms]	48,683
3.	#1 AND #2	(*Cryptosporidium*[MeSH Terms] OR "*Cryptosporidium parvum*"[MeSH Terms] OR Coccidia[MeSH Terms] OR Cryptosporidiidae[MeSH Terms] OR Cryptosporidiosis[MeSH Terms] OR *Cryptosporidium*[Text Word] OR "*Cryptosporidium parvum*"[Text Word] OR Coccidia[Text Word] OR Cryptosporidiidae[Text Word] OR Cryptosporidiosis[Text Word]) AND (Thailand[Text Word] OR Thailand[MeSH Terms] OR Siam[Text Word] OR Siam[MeSH Terms])	189

Embase 20 March 2025

No.	Key concept	Search terms	Results
1.	*Cryptosporidium*	*Cryptosporidium*/exp OR "*Cryptosporidium parvum*"/exp OR Coccidia/exp OR Cryptosporidiidae/exp OR Cryptosporidiosis/exp OR *Cryptosporidium*:ti,ab,kw,de OR "*Cryptosporidium parvum*":ti,ab,kw,de OR Coccidia:ti,ab,kw,de OR Cryptosporidiidae:ti,ab,kw,de OR Cryptosporidiosis:ti,ab,kw,de	54,793
2.	Thailand	Thailand:ti,ab,kw,de OR Thailand/exp OR Siam:ti,ab,kw,de OR Siam/exp	56,824
3.	#1 AND #2	(*Cryptosporidium*/exp OR "*Cryptosporidium parvum*"/exp OR Coccidia/exp OR Cryptosporidiidae/exp OR Cryptosporidiosis/exp OR	267

		Cryptosporidium:ti,ab,kw,de OR "*Cryptosporidium parvum"*:ti,ab,kw,de OR Coccidia:ti,ab,kw,de OR Cryptosporidiidae:ti,ab,kw,de OR Cryptosporidiosis:ti,ab,kw,de) AND (Thailand:ti,ab,kw,de OR Thailand/exp OR Siam:ti,ab,kw,de OR Siam/exp)	

Scopus 20 March 2025

No.	Key concept	Search terms	Results
1.	*Cryptosporidium*	TITLE-ABS-KEY (cryptosporidium OR "Cryptosporidium parvum" OR coccidia OR cryptosporidiidae OR cryptosporidiosis)	24,352
2.	Thailand	TITLE-ABS-KEY (thailand OR siam)	132,015
3.	1 AND 2	(TITLE-ABS-KEY (cryptosporidium OR "Cryptosporidium parvum" OR coccidia OR cryptosporidiidae OR cryptosporidiosis)) AND (TITLE-ABS-KEY (thailand OR siam))	131

Journal@Ovid 20 March 2025

No.	Key concept	Search terms	Results
1.	*Cryptosporidium* AND Thailand	(Cryptosporidium OR "Cryptosporidium parvum" OR Coccidia OR Cryptosporidiidae OR Cryptosporidiosis) AND (Thailand OR Siam) {Including Limited Related Terms}	1165

Nursing & Allied Health Premium 21 March 2025

No.	Key concept	Search terms	Results
1.	*Cryptosporidium* AND Thailand	(*Cryptosporidium* OR "*Cryptosporidium parvum"* OR Coccidia OR Cryptosporidiidae OR Cryptosporidiosis) AND (Thailand OR Siam)	382

Google Scholar, 19 March 2025

No.	Key concept	Search terms	Results
1.	*Cryptosporidium* AND Thailand	*Cryptosporidium* AND Thailand	The first 200 results

Google Scholar (n = 200), Date 19 March 2025

- Not related (n = 168)
- Related (n = 32)

    - Included (n = 3)
    - Excluded (n = 29)
        - Duplicated with selected articles from main databases (21)
        - No case of *Cryptosporidium* infection (3)
        - Review (1)
        - Not in Thailand (1)
        - Not Thai participants (1)
        - Comparative studies enrolled *Cryptosporidium*-positive samples (2)

Thai-Journal Citation Index, 9 January 2025

No.	Key concept	Search terms	Results
1.	*Cryptosporidium* AND Thailand	*Cryptosporidium*	33

TCI selection (n = 33), Date 19 March 2025

- Included (n= 1)
- Excluded (n = 32)
    - Not in Thailand (n = 9)
    - Animal samples (n = 5)
    - Assay development (n = 5)
    - Environment samples (n = 4)
    - Duplicated with Google Scholar (n = 3)
    - In vitro study (n = 2)
    - Review (n = 2)
    - Case report (n = 1)
    - No record (n = 1)
