# Supplementary material for: Prevalence of Cryptosporidium Infections in Thailand and Its Association with HIV and Diarrhea: A Systematic Review and Meta-Analysis
Source: Med Sci (Basel). 2025 Aug 26;13(3):156. doi: 10.3390/medsci13030156 (PMC12452679; doi:10.3390/medsci13030156)
Supplement: Supplementary file 1 [file medsci-13-00156-s001.zip › Table S4. Meta-regression and subgroup analysis.pdf]

Table S4. Meta-regression and subgroup analysis of the pooled prevalence of *Cryptosporidium* infections in Thailand.

1. Meta-regression analysis of the pooled prevalence of *Cryptosporidium* infections in Thailand.

**Covariates**	**tau^2**	**Test for residual heterogeneity, *P* value**	**Residual heterogeneity I^2 (%)**	**H^2**	**Test of moderators, *P* value**	**Number of studies (subsets)**
Publication years	2.9473	< 0.001	97.99	49.69	0.7526	35
Study design	2.9413	< 0.001	98.01	50.21	0.9941	35
Regions of Thailand	2.1890	< 0.001	97.47	39.54	0.0648	35
Types of participants	0.5481	< 0.001	89.69	9.7	< 0.001	35
Age groups	1.8820	< 0.001	96.43	28.02	0.0006	35
Male percentage	2.8083	< 0.001	97.75	44.37	0.0069	23
Diarrheal status	2.1417	< 0.001	96.99	33.18	< 0.001	35
Detection method for *Cryptosporidium*	2.8953	< 0.001	98.03	50.64	0.7929	35

2. Subgroup analysis of the pooled prevalence of *Cryptosporidium* infections in Thailand.

Pooled prevalence	Subgroup	Test for subgroup difference	Pooled prevalence (%) [95% CI]	I^2 (%)	Number of studies (subsets)
Overall			4.70 [0.03; 0.08]	97.1	35
Publication years		0.81			
	Before 2000		5.06 [2.47; 10.09]	95.4	11
	2000–2022		4.47 [2.08; 9.34]	97.3	24
Study designs		0.98			
	Cross-sectional study		4.59 [2.27; 9.06]	96.7	24
	Prospective observational study		5.05 [2.61; 9.54]	82.4	6
	Retrospective observational study		4.68 [0.67; 26.32]	98.9	5
Parts of Thailand		< 0.0001			
	Central Thailand		6.08 [3.36; 10.8]	97.6	27
	Western Thailand		0.51 [0.08; 3.26]	91.8	3
	Northern Thailand		11.1 [8.56; 14.4]	N/A	1
	Northeastern Thailand		6.41 [3.48; 11.5]	N/A	1
	Southern Thailand		5.74 [2.76; 11.6]	N/A	1
	Northeastern, Southern, eastern Thailand		0.14 [0.02; 1.01]	N/A	1
	Not specified		12.00 [5.49; 24.24]	N/A	1
Age groups of participants		0.0014			
	Children		3.19 [1.80; 5.60]	87.9	9
	Adults		18.27 [8.05; 36.3]	96.1	9
	Mixed age groups		1.43 [0.40; 4.90]	95.4	9
	Not specified		5.47 [2.40; 12.0]	95.3	8
Participants		< 0.0001			
	HIV-infected patients		16.33 [10.93; 23.69]	94.4	16
	HIV-infected and -uninfected patients		4.02 [3.51; 4.61]	0.0	4
	Participants suspected for parasite infections		0.44 [0.11; 1.66]	95.8	3
	Children with diarrhea		3.27 [1.18; 8.73]	87.1	3
	HIV--seropositive and -seronegative patients		6.12 [3.83; 9.62]	0.0	2
	Monks or nuns		1.47 [0.48; 4.46]	N/A	1

	Orphanage children		8.29 [5.22; 12.93]	N/A	1
	Villagers and school children		0.14 [0.02; 1.01]	N/A	1
	Adult patients		0.51 [0.13; 2.03]	N/A	1
	School children		0.39 [0.06; 2.74]	N/A	1
	Pre-school children		3.39 [2.09; 5.46]	N/A	1
	Refugees		0.11 [0.03; 0.43]	N/A	1
Detection method for *Cryptosporidium*		0.54			
	Standard method		4.61 [2.41; 8.64]	97.5	29
	- PCR method		5.99 [0.71; 36.3]	96.5	6
	- Non-PCR methods		5.41 [3.18; 9.05]	97.0	33
	Non-standard method		10.56 [2.21; 38.16]	89.0	2
	Not specified		3.82 [1.29; 10.77]	80.7	4
Diarrheal status		0.0002			
	Diarrhea		7.94 [4.11; 14.79]	85.8	10
	Diarrhea and non-diarrhea		7.19 [3.70; 13.48]	97.9	17
	Non-diarrhea		0.14 [0.02; 1.01]	N/A	1
	Not specified		1.16 [0.25; 5.22]	97.2	7

N/A, not assessed

3. Subgroup analysis of the pooled prevalence of *Cryptosporidium* infections in several provinces of Thailand

Regions of Thailand	Province	Pooled prevalence (%) [95% CI]	I^2 (%)	Number of studies (subsets)
Overall		4.70 [0.0268; 0.0813]	97.1	35
Central Thailand		6.08 [3.36; 10.76]	97.6	27
	Bangkok	2.86 [1.46; 5.50]	95.1	17
	Nonthaburi	8.57 [4.07; 17.15]	92.6	4
	Lop Buri	32.35 [25.03; 40.65]	0.0	2
	Bangkok, Nonthaburi	29.54 [14.19; 51.53]	97.3	4
Western Thailand		0.51 [0.08; 3.26]	91.8	3
	Ratchaburi	0.39 [0.06; 2.74]	N/A	1
	Kanchanaburi	3.39 [2.09; 5.46]	N/A	1
	Thailand-Burma border	0.11 [0.03; 0.43]	N/A	1
Northern Thailand	Phayao	11.14 [8.56; 14.36]	N/A	1
Northeastern Thailand	Khon Kaen	6.41 [3.48; 11.50]	N/A	1
Southern Thailand	Songkhla	5.74 [2.76; 11.55]	N/A	1
Multi-regions	Chiang rai, Nan, Tak, Ratchaburi, Loei, Chumphon, Sa Kaeo	0.14 [0.02; 1.01]	N/A	1
Not specified	Thai-Kampuchean border	12.00 [5.49; 24.24]	N/A	1

N/A, not assessed.
