# Supplementary material for: Prevalence of Cryptosporidium Infections in Thailand and Its Association with HIV and Diarrhea: A Systematic Review and Meta-Analysis
Source: Med Sci (Basel). 2025 Aug 26;13(3):156. doi: 10.3390/medsci13030156 (PMC12452679; doi:10.3390/medsci13030156)
Supplement: Supplementary file 1 [file medsci-13-00156-s001.zip › Table S5. Meta-regression and subgroup analysis.pdf]

Table S5. Subgroup analysis of the pooled odds ratio of having diarrhea among participants with and without *Cryptosporidium* infections in Thailand.

**Subgroups**	**Subgroup**	**Test for subgroup difference**	**Pooled odds ratio [95% CI]**	I^2 **(%)**	**Number of studies**
Overall			2.00 [0.67; 5.99]	70	8
Parts of Thailand		0.0004			
	Central Thailand		3.71 [1.17; 11.8]	39.1	5
	Western Thailand		0.37 [0.17; 0.84]	0.0	2
	Northeastern Thailand		5.07 [1.25; 20.5]	N/A	1
Province		< 0.0001			
	Bangkok		2.23 [0.86; 5.79]	0.0	4
	Kanchanaburi		0.37 [0.17; 0.84]	0.0	2
	Nonthaburi		82.1 [4.96; 1358.3]	N/A	1
	Khon Kaen		5.07 [1.25; 20.5]	N/A	1
Age groups of participants		0.01			
	Children		0.89 [0.25; 3.15]	60.7	4
	Mixed age groups		82.0 [4.96; 1358.2]	N/A	1
	Not specified		2.65 [1.01; 6.95]	0.0	3
Participants		0.001			
	HIV-infected patients		2.50 [0.66; 9.55]	0.0	2
	Pre-school children		0.37 [0.17; 0.84]	0.0	2
	Children with diarrhea		22.3 [1.65; 301.9]	40.0	2
	HIV-seropositive and -seronegative patients		5.07 [1.25; 20.5]	N/A	1
	HIV-infected and -uninfected patients		1.47 [0.32; 6.83]	N/A	1
Detection method for *Cryptosporidium*		N/A			
	Standard method		2.00 [0.67; 5.99]	70.0	8

N/A, not assessed
