# Supplementary material for: Prevalence of Cryptosporidium Infections in Thailand and Its Association with HIV and Diarrhea: A Systematic Review and Meta-Analysis
Source: Med Sci (Basel). 2025 Aug 26;13(3):156. doi: 10.3390/medsci13030156 (PMC12452679; doi:10.3390/medsci13030156)
Supplement: Supplementary file 1 [file medsci-13-00156-s001.zip › Table S6. Influential analysis.pdf]

Table S6. Influential analysis (common effect model)

	proportion	95%-CI	p-value	tau^2	tau	I^2
Omitting Anekthananon et al., 2004	0.0325	[0.0303; 0.0348]		3.0402	1.7436	97.1%
Omitting Chokephaibulkit et al., 2001	0.0327	[0.0305; 0.0351]		3.0320	1.7413	97.1%
Omitting Inpankaew et al., 2007	0.0329	[0.0307; 0.0352]		2.9766	1.7253	97.1%
Omitting Janoff et al., 1990	0.0323	[0.0301; 0.0346]		3.0309	1.7410	97.2%
Omitting Jantanavivat et al., 1991	0.0353	[0.0329; 0.0379]		2.9941	1.7303	96.8%
Omitting Jirapinyo et al., 1993	0.0331	[0.0309; 0.0354]		2.9686	1.7230	97.1%
Omitting Jongwutiwes et al., 1990	0.0329	[0.0307; 0.0353]		3.0263	1.7396	97.1%
Omitting Leelayoova et al., 2001	0.0325	[0.0303; 0.0348]		3.0350	1.7421	97.2%
Omitting Manatsathit et al., 1996	0.0324	[0.0302; 0.0348]		2.9629	1.7213	97.1%
Omitting Moolasart et al., 1995	0.0322	[0.0300; 0.0345]		3.0286	1.7403	97.2%
Omitting Mungthin et al., 2003	0.0325	[0.0304; 0.0349]		3.0398	1.7435	97.1%
Omitting Nordlander et al., 1990	0.0326	[0.0304; 0.0349]		3.0112	1.7353	97.1%
Omitting Nuchjangreed et al., 2008	0.0323	[0.0301; 0.0346]		2.9075	1.7051	97.1%
Omitting Nuchprayoon et al., 2002	0.0440	[0.0411; 0.0471]		2.5050	1.5827	96.8%
Omitting Pinlaor et al., 2005	0.0325	[0.0303; 0.0349]		3.0371	1.7427	97.2%
Omitting Prasertbun et al., 2019	0.0337	[0.0314; 0.0361]		2.6953	1.6417	97.1%
Omitting Punpoowong et al., 1998	0.0327	[0.0305; 0.0350]		3.0185	1.7374	97.2%
Omitting Saksirisampant et al., 2002	0.0321	[0.0299; 0.0344]		3.0053	1.7336	97.1%
Omitting Saksirisampant et al., 2009	0.0316	[0.0294; 0.0339]		2.8588	1.6908	97.0%
Omitting Sannella et al., 2019	0.0286	[0.0265; 0.0308]		2.5685	1.6027	95.8%
Omitting Srisuphanunt et al., 2011	0.0316	[0.0294; 0.0339]		2.9459	1.7164	97.1%
Omitting Srisuphanunt et al., 2008	0.0320	[0.0298; 0.0343]		2.9842	1.7275	97.1%
Omitting Supcharassaeng et al., 2011	0.0332	[0.0310; 0.0356]		2.8642	1.6924	97.1%
Omitting Sutthikornchai et al., 2021	0.0331	[0.0308; 0.0354]		2.8458	1.6869	97.1%
Omitting Suwancharoen et al., 2018	0.0312	[0.0290; 0.0335]		3.0153	1.7365	97.1%
Omitting Thamlikitkul et al., 1987	0.0346	[0.0323; 0.0371]		2.8601	1.6912	97.0%

Omitting Uga et al., 1998	0.0326 [0.0304; 0.0350]	3.0370	1.7427	97.2%
Omitting Viriyavejakul et al., 2009	0.0323 [0.0301; 0.0346]	2.9587	1.7201	97.1%
Omitting Wanachiwanawin et al., 2002	0.0327 [0.0305; 0.0351]	3.0287	1.7403	97.1%
Omitting Wanachiwanawin et al., 1998	0.0319 [0.0297; 0.0342]	2.9232	1.7097	97.1%
Omitting Waywa et al., 2006	0.0311 [0.0288; 0.0337]	3.0406	1.7437	96.9%
Omitting Waywa et al., 2001	0.0308 [0.0287; 0.0331]	2.9627	1.7212	97.0%
Omitting Webster et al., 2022	0.0354 [0.0330; 0.0379]	2.5927	1.6102	97.1%
Omitting Wiwanitkit et al., 2001	0.0327 [0.0306; 0.0351]	3.0165	1.7368	97.1%
Omitting Wongstitwilairoong et al., 2007	0.0327 [0.0305; 0.0351]	3.0342	1.7419	97.1%
Pooled estimate	0.0327 [0.0306; 0.0351]	2.9412	1.7150	97.1%
